# Virtual reality and behaviour management in paediatric dentistry: a systematic review

**DOI:** 10.1186/s12903-023-03595-7

**Published:** 2023-12-12

**Authors:** Diana Xavier de Barros Padilha, Nélio Jorge Veiga, Anna Carolina Volpi Mello-Moura, Patrícia Nunes Correia

**Affiliations:** 1 Faculty of Dental Medicine, Universidade Católica Portuguesa, 3504-505 Viseu, Portugal; 2 Centre for Interdisciplinary Research in Health (CIIS), Universidade Católica Portuguesa, 3504-505 Viseu, Portugal

**Keywords:** Virtual reality, Paediatric dentistry, Distraction, Dental anxiety, Pain, Behaviour management

## Abstract

**Background:**

Virtual reality (VR) has emerged as an innovative tool in medicine and dentistry, improving anxiety and pain management in children. The immersive and interactive environments of VR technology facilitate positive engagement of young patients during dental procedures via distraction, potentially reducing anxiety levels and improving treatment experience. The aim of this review was to provide current evidence-based guidance on the usage of VR in the clinical practice of paediatric dentistry.

**Methods:**

A systematic review was conducted according to the PRISMA guidelines with the following research question using the PICO format: Does VR (I) effectively manage anxiety and pain (O) during a paediatric dental consultation (P) compared to alternative behavioural control techniques (C)? PubMed/Medline®, SCOPUS and Web of Science databases were searched and analysed.

**Results:**

A total of 22 randomised control trials were included in this review. These studies have shown that VR is a highly effective method of behaviour management, successfully alleviating pain and anxiety in children during dental treatment, surpassing traditional tools. Selected studies included participants with a large age range and dental procedures varied greatly, from first consultations to infiltration of local anaesthetic and other invasive procedures. VR was mostly used during treatment delivery and different immersive VR techniques were considered. Behaviour, anxiety and pain scales were used to determine efficacy and patient satisfaction.

**Conclusions:**

VR offers an engaging and immersive experience, effectively diverting patients' attention away from the clinical environment, fostering a positive and enjoyable treatment experience. However, it is important to acknowledge the limitations of existing studies and the need for further research to enhance the understanding of VR's full potential in paediatric dentistry.

**Supplementary Information:**

The online version contains supplementary material available at 10.1186/s12903-023-03595-7.

## Background

Fear and apprehension regarding dental treatment are the most significant factors negatively impacting the daily clinical work of paediatric dentists [1]. Dental appointments can trigger anxiety and pain, leading to treatment avoidance or refusal which can worsen the patient's overall health condition. Factors contributing to dental fear and anxiety, as noted by Dahlander et al. 2019, include parental previous negative experiences, lack of information about the treatment, type of treatment, and the dental environment itself [2].

For anxious children, distraction can be an effective method of diverting the patient's attention from procedures that are considered unpleasant [3]. A variety of distraction techniques are employed to mitigate anxiety and enhance the dental experience for young patients [4]. Audiovisual distractions, such as tablet devices and smartphones, play a crucial role in engaging children and diverting attention in their daily lives [5, 6], and are widely accepted by children and parents during medical procedures [7].

With the rapid advancement of technology, audiovisual glasses emerged as a significant development in distraction techniques. These glasses allowed videos to be displayed in a two-dimensional format (2D), providing an enhanced visual experience for paediatric patients. These progressed to virtual reality glasses which unlike the 2D glasses enable the display of interactive content in a three-dimensional format (3D) [8]. This advancement immerses patients in a more realistic and engaging virtual environment, offering a heightened sense of presence and interactivity during dental procedures [9, 10].

Virtual reality creates an artificial environment that mimics the real world, allowing users to experience an alternate world [11, 12]. The virtual experience provides multi-sensory information through synchronization between the head-mounted display helmet (provides an image with a sense of space and depth), motion sensors, headphones and joysticks, for a fully immersive simulation [13].

During the past two decades, virtual reality technologies have been used for entertainment [14], education [15], training [16], research [17] and much more. Virtual reality technology is becoming increasingly accessible and powerful, and the potential uses are virtually limitless [18, 19].

In the medical field, as an effective and efficient tool to prevent emotional disorders such as anxiety [20] and physical impairments in rehabilitation processes [21], and lately as a method of pain reduction [22, 23]. Like doctors, nurses and dentists in training, allowing them to experience real medical situations before treating real patients [24, 25].

In dentistry, although not yet widespread, VR has proven to be a beneficial tool for clinical practice in several specialties [26]. From student training [25, 27] to predicting surgical complications [28], doctors can use virtual reality technology to show their patients the expected results before undergoing the procedure [29].

In paediatrics, VR can be effective for oral hygiene education and maintenance, reduction of anxiety and pain [30, 31].

Also, using virtual reality devices during consultation/ treatment visits allow patients to virtually experience the entire scenario before the commencement of the actual procedure. Thus, enabling a better understanding of the treatment and allowing fears to be confronted in a safe and controlled environment [32, 33].

In recent years, virtual reality has gained popularity in clinical research studies as an innovative technique for modulating paediatric behaviour [19, 27, 34]. According to McCaul et al. 1992, the perception and attention to pain play crucial roles in pain experience. VR does not directly impact the pathophysiological mechanisms of pain but rather focuses on modifying patients' perception and attention to pain [35].

While VR in dentistry is not yet widely adopted, it has demonstrated considerable benefits across various specialties [26]. This systematic review aims to explore the current available evidence on the use of VR for controlling pain and anxiety in children during dental consultations.

## Methodology

This systematic review adhered to the PRISMA (Preferred Reporting Items for Systematic Reviews and Meta-Analyses) guidelines [36], and the research question was formulated using the PICO (Population, Intervention, Comparison, Outcome) format. The objective of this review was to investigate the effectiveness of virtual reality (VR) in controlling anxiety and pain during dental appointments in the paediatric population (P), in comparison to other behavioural control techniques (C).

The review protocol was registered in the International Prospective Register of Systematic Reviews (PROSPERO) with the registration number CRD4202340967. A comprehensive literature search was conducted in January 2023, using the PubMed/Medline®, SCOPUS and Web of Science databases. The search results were exported to the Parsifal bibliography manager software, where duplicates were removed and articles were selected based on the defined objective and criteria (Table 1).

**Table 1:** Inclusion and exclusion criteria

Inclusion criteria	Exclusion criteria
Studies designed as randomized clinical trials (RCTs), experimental and case–control study designs with a full-text report	Systematic/narrative reviews and meta-analyses, case reports, case series
Full text articles from 2003–2023	Studies without a full-text report
Children under 18, female or male	Patients with any visual and/or hearing impairment
Use of virtual reality interventions to help ease anxiety and pain during dental procedures	Use of 2D devices

The search strategy employed a combination of subject heading terms, keywords and text words, utilizing Boolean operators such as 'OR' and 'AND' (Tables 2 and 3).

**Table 2:** Search strategy in PubMed

Concepts	PUBMED
**#1**	“VR"[All Fields] OR "virtual reality"[All Fields] OR "augmented reality"[All Fields] OR "AR"[All Fields] OR "mixed reality"[All Fields] OR "Audiovisual distraction"[All Fields] OR "audiovisual"[All Fields] OR "Audiovisual Aids"[All Fields] OR "headset*"[All Fields] OR "vr headset*"[All Fields] OR "virtual reality headset"[All Fields] OR "AR headset"[All Fields] OR "augmented reality headset"[All Fields] OR "Artificial intelligence"[All Fields] OR "VR goggles"[All Fields] OR "virtual reality goggles"[All Fields] OR "AR goggles"[All Fields] OR "augmented reality goggles"[All Fields] OR "Virtual Reality Exposure Therapy"[All Fields] OR "VR Exposure Therapy"[All Fields] OR "Augmented Reality Exposure Therapy"[All Fields] OR "Virtual Reality Exposure Therapy"[MeSH Terms] OR "Audiovisual Aids"[MeSH Terms] OR "augmented reality"[MeSH Terms]
**#2**	"child, preschool"[MeSH Terms] OR "preschool child"[All Fields] OR "paediatric population"[All Fields] OR "paediatric patient*"[All Fields] OR "child"[MeSH Terms] OR "child*"[All Fields] OR "adolescent"[MeSH Terms] OR "adolescen*"[All Fields] OR "pre schooler*"[All Fields] OR "youth"[All Fields] OR "teenager*"[All Fields] OR "teen*"[All Fields] OR "preteen*"[All Fields] OR "pre teen*"[All Fields] OR "pediatrics"[MeSH Terms] OR "paediatric*"[All Fields] OR “Autistic Disorder”[MeSH Terms] OR “Autism”[All Fields] OR “Down Syndrome”[MeSH Terms] OR “Down Syndrome”[All Fields]
**#3**	"Pain"[All Fields] OR "Pain Management"[All Fields] OR "dental pain"[All Fields] OR "Pain Perception"[All Fields] OR "Anxiety"[All Fields] OR "Dental anxiety"[All Fields] OR "anticipatory anxiety"[All Fields] OR "fear"[All Fields] OR "stress"[All Fields] OR "Dental anxiety"[MeSH Terms] OR "Pain Management"[MeSH Terms] OR "Pain"[MeSH Terms] OR "Pain Perception"[MeSH Terms]
**#4**	"dental care"[All Fields] OR "dental procedure*"[All Fields] OR "dental operation*"[All Fields] OR "dental appointment*"[All Fields] OR "dental treatment*"[All Fields] OR “dent*”[All Fields] OR “dental hospital*”[All Fields] OR “dentistry”[MeSH Terms] OR "dental care"[MeSH Terms]
**#5**	#1 AND #2 AND #3 AND #4

**Table 3:** Search strategy in Scopus and Web of Science

Concepts	SCOPUS
**#1**	“VR" OR "virtual reality" OR "augmented reality" OR "AR" OR "mixed reality" OR "Audiovisual distraction" OR "audiovisual" OR "Audiovisual Aids" OR "headset*" OR "vr headset*" OR "virtual reality headset" OR "AR headset" OR "augmented reality headset" OR "Artificial intelligence" OR "VR goggles" OR "virtual reality goggles" OR "AR goggles" OR "augmented reality goggles" OR "Virtual Reality Exposure Therapy" OR "VR Exposure Therapy" OR "Augmented Reality Exposure Therapy"
**#2**	"preschool child" OR "paediatric population" OR "paediatric patient*" OR "child*" OR "adolescen*" OR "pre schooler*" OR "youth" OR "teenager*" OR "teen*" OR "preteen*" OR "pre teen*" OR "paediatric*" OR "Autistic Disorder" OR "Autism" OR "Down Syndrome"
**#3**	"Pain" OR "Pain Management" OR "dental pain" OR "Pain Perception" OR "Anxiety" OR "Dental anxiety" OR "anticipatory anxiety" OR "fear" OR "stress"
**#4**	"dental care" OR "dental procedure*" OR "dental operation*" OR "dental appointment*" OR "dental treatment*" OR “dent*” OR “dental hospital*”
**#5**	#1 AND #2 AND #3 AND #4
**Concepts**	**WEB OF SCIENCE**
**#1**	(VR) OR (virtual reality) OR (augmented reality) OR (AR) OR (mixed reality) OR (Audiovisual distraction) OR (audiovisual) OR (Audiovisual Aids) OR (headset*) OR (vr headset*) OR (virtual reality headset) OR (AR headset) OR (augmented reality headset) OR (Artificial intelligence) OR (VR goggles) OR (virtual reality goggles) OR (AR goggles) OR (augmented reality goggles) OR (Virtual Reality Exposure Therapy) OR (VR Exposure Therapy) OR (Augmented Reality Exposure Therapy)
**#2**	(preschool child) OR (paediatric population) OR (paediatric patient*) OR (child*) OR (adolescen*) OR (pre schooler*) OR (youth) OR (teenager*) OR (teen*) OR (preteen*) OR (pre teen*) OR (paediatric*) OR (Autistic Disorder) OR (Autism) OR (Down Syndrome)
**#3**	(Pain) OR (Pain Management) OR (dental pain) OR (Pain Perception) OR (Anxiety) OR (Dental anxiety) OR (anticipatory anxiety) OR (fear) OR (stress)
**#4**	(dental care) OR (dental procedure*) OR (dental operation*) OR (dental appointment*) OR (dental treatment*) OR (dent*) OR (dental hospital*)
**#5**	#1 AND #2 AND #3 AND #4

Two independent researchers (DP and PC) performed the search and screening procedure for this systematic review, following the predetermined inclusion and exclusion criteria. In the event of any disagreement between the researchers, a third researcher (AM) was consulted to resolve it.

To assess agreement and reliability between researchers, Cohen's Kappa coefficient was employed. The coefficient ranges from -1 to 1, with values closer to 1 indicating higher agreement between reviewers and values closer to -1 indicating greater disagreement.

Data extracted included author, year, study design, sample size, age, dental procedure, intervention used, timing of intervention, control/comparison groups, outcomes and outcome measures.

The quality assessment of the included studies was conducted using the Newcastle Ottawa Scale (NOS), (Additional file 1: Appendix 1) [37].

## Results

### Study selection

**Fig. 1:**
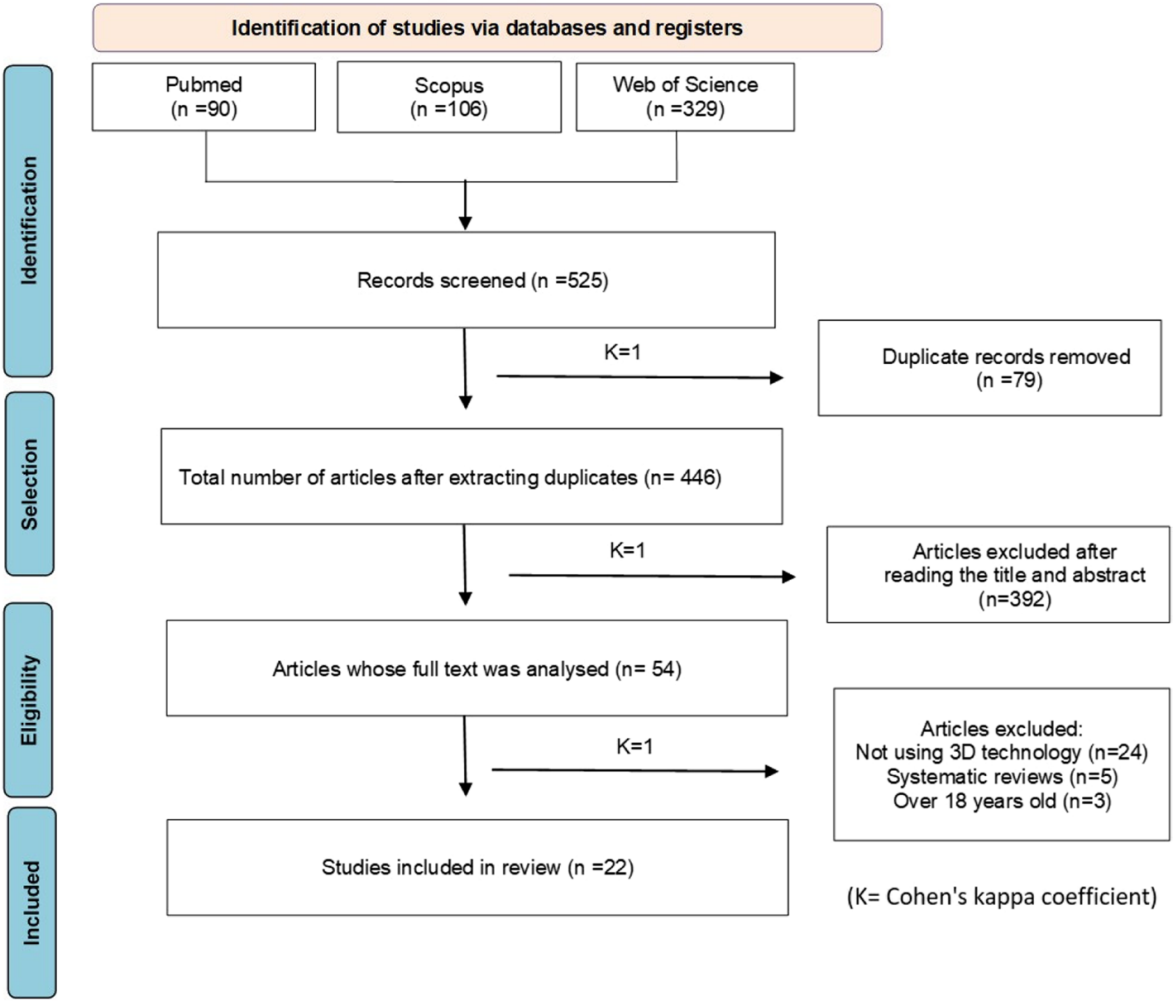
PRISMA search strategy

The search queries yielded a total of 525 abstracts from three different databases. After removing 79 duplicate articles, 446 unique abstracts remained. Upon reviewing the titles and abstracts, 392 records were deemed irrelevant and excluded. Subsequently, 54 articles were selected for full-text analysis. Among these, 32 articles were excluded as they utilized audiovisual glasses without 3D immersion. Ultimately, 22 articles were considered suitable for inclusion in this systematic review (Fig. 1).

As stated in Fig. 1, inter-rater agreement, was determined and a kappa value of 1 was obtained during the selection process, indicating an excellent agreement.

### Study characteristics

This analysis included 22 studies conducted in various countries, including Turkey, Iran, India, China, Jordan, Spain, Syria, Italy and Indonesia. All of these studies were randomised control trials [38–59]. Tables 4, 5 and 6 offer a comprehensive overview of the studies characteristics: authors, publication date, country, study type, participants number and ages and study outcomes. The VR equipment utilized in the studies was also included. Out of the 22 studies, two (9%) investigated the use of the Oculus Go device, which is a standalone virtual reality headset [41, 58]. Additionally, four studies (18%) selected a VR Box device and two chose a HTC device, an all-in-one headset [38, 42, 45, 55, 57, 59]. In four studies, the VR device utilized was not specified [39, 52, 53, 56]. The remaining studies, as shown in Tables 4, 5 and 6, employed various other devices that were not replicated in other research. Overall, these devices demonstrated high resolution, light weight and compact size.

**Table 4:** Summary of results (A1-A8)

Study ID	Authors Year Country	Study Design	Participants Number Age	Results	Dependent Variables	Control Groups	VR Equipment	VR Content
**A1** [38]	Ozukoc et al. [38] 2020 Turkey	RCT	23 10–12 years	VR had best result across all MIH severity levels	CPMAS	Vs control	Preo VR Box	Video games (InCell VR Cardboard Android 1.4.3)
**A2** [39]	Baniebrahimi et al. [39] 2022 Iran	RCT	42 5–8 years	Anxiety significantly lower in the VR	FBRS, MCDAS, FIS	Vs game apps	Not specified	Not specified
**A3** [40]	Shetty et al. [40] 2019 India	RCT	120 58 years	Pain and anxiety significant reduction in VR group	SCARED, MCDAS, Salivary cortisol levels, WBFS	Vs control	i-glasses 920HR, Ilixco Inc	Child´s favorite cartoon show
**A4** [41]	Kumari et al. [41] 2021 India	RCT	200 6–12-years	Immersive group had best results	FBRS, MCDAS, WBFS, VAS	Vs Non-immersive VR	Oculus Go Standalone	Immersive VR (videogame) Non-immersive VR (cartoon movies)
**A5** [42]	Ran et al. [42] 2021 China	RCT	120 4–8 years	VR significantly reduced the anxiety and pain	FBRS, WBFS**,** CFSS-DS	Vs control	HTC’s VIVE	Undersea scenes
**A6** [43]	Alshatrat et al. [43] 2022 Jordan	RCT	54 5–12 years	VR was found to be an effective distraction	WBFS, FLACC, VAS	Vs control	iWear Vuzix®	Videos of their preference
**A7** [44]	Gomez-Polo et al. [44] 2021 Spain	RCT	80 5–10 years	VR effectively managed anxiety and behavior	FBRS**,** FIS	Vs control	Zeiss Cinemizer (Carl Zeiss AG)	Cartoons or children’s movies of their preference
**A8** [45]	Du et al. [45] 2022 China	RCT	128 4–9 years	VR significantly reduced anxiety/pain perception	Houpt Scale, WBFS, CFSS-DS, SSQ	Vs control	HTC	A magic virtual world of their preference

**Table 5:** Summary of results (A9-A17)

Study ID	Authors Year Country	Study Design	Participants Number Age	Results	Dependent Variables	Control Groups	VR Equipment	VR Content
**A9** [46]	Aditya et al. [46] 2021 India	RCT	60 6–9 years	VR significantly reduced the anxiety and pain	VPT, Pulse oximeter	vs control vs Fidget spinner vs Kaleidoscope	MI VR Headset	Cartoon episode
**A10** [47]	Nuvvula et al. [47] 2015 India	RCT	90 7 10 years	3D group had higher levels of satisfaction	FBRS, Houpt Scale, MCDAS, Pulse rate	vs control vs Music	Vuzix Eyewear Wrap 920	Movies
**A11** [48]	Murali et al. [48] 2021 India	RCT	75 5–8 year	VR had best results	FIS	vs control	Virtual private theater system	Not specified
**A12** [49]	Felemban et al. [49] 2021 Saudi Arabia	RCT	50 6–12 years	VR helped to overcome dental anxiety	BAS**,** Pulse oximeter, WBFS, FLACC	vs regular screen	LG 360, LG Electronics	Chosen video
**A13** [50]	Al-Halabi et al. [50] 2018 Syria	RCT	102 6 -10 years	Tablet had the bests results anxiety and pain	BAS**,** Pulse rate, WBFS	vs control vs tablet	BlackBug™	Cartoon episode
**A14** [51]	Buldur et al. [51] 2021 Turkey	RCT	78 7–11 years	VR significantly reduced pain and anxiety	FBRS, Pulse rate, WBFS	vs control	PlayStation 4 VR, Sony Inc	Chosen animated films or cartoons
**A15** [52]	Sharma et al. [52] 2021 India	RCT	97 4–8 years	VR effectively managed anxiety and behaviour	FBRS, FLACC	vs control	Not specified	Age appropriate videos according to subjects’ choice
**A16** [53]	Khan et al. [53] 2019 India	RCT	100 4–10 year	VR made children less anxious and more cooperative	Pulse rate	vs control	Not specified	Cartoon clips and visual reality films
**A17** [54]	Atzori et al. [54] 2018 Italy	RCT	5 7–17 years	VR increase fun during dental procedures	0–10 graphic rating scale	vs control	Oculus Rift DK2 and CV1	SnowWorld VR software

**Table 6:** Summary of results (A18-A22)

Study ID	Authors Year Country	Study Design	Participants Number Age	Results	Dependent Variables	Control Groups	VR Equipment	VR Content
**A18** [55]	Niharika et al. [55] 2018 India	RCT	40 4–8 years	VR significantly reduced the anxiety and pain	WBFS, MCDAS, Pulse rate, oximeter	vs control	Google VR Box and Anti Tank VR 3D Glasses	Cartoon series “Doreman”
**A19** [56]	Pande et al. [56] 2020 India	RCT	60 5–8 years	VR was most effective in reducing dental fear/anxiety	Pulse rate, FIS	vs control vs audio vs smartphone app	Not specified	Patients´favorite cartoon
**A20** [57]	Greeshma et al. [57] 2021 India	RCT	90 6/8 years	Children were most relaxed in VR group,	FIS, Pulse rate, oximeter	vs control vs audio	Ocular (?) VR Box	3D video with audio (VR roller coaster)
**A21** [58]	Zaidman et al. [58] 2022 Israel	RCT	29 4–12 years	VR decreased pain during rubber dam placement	WBFS, MBPS	vs control	Oculus Go	3 types Two cartoon series and a children’s show, average screening time 30 min
**A22 **[59]	Kaswindiarti et al. [59] 2022 Indonesia	RCT	120 5–8 years	Pain/anxiety decrease significantly using VR	WBFS, Salivary cortisol levels	vs control	VR Box	cartoon SpongeBob SquarePants screentime three-four minutes

For each study, the risk of bias was assessed with the NOS scale (Additional file 1: Appendix 2). Two studies had a very low risk of bias [50, 55], thirteen studies had a moderate risk of bias [38–42, 47, 49, 51, 52, 55–58] and seven had a high risk of bias [43–46, 48, 53, 54].

The studies investigated the effectiveness of different immersive VR techniques, compared to various behavioural control techniques in paediatric dentistry: passive distraction, the tell-show-do technique [38, 40, 42–48, 51, 53–59], including digital screen and audio-visual distraction [39, 41, 49, 50, 52, 56]. The total number of participants involved in the studies was 2,558, with most studies focusing on children aged between 5 and 12 years. However, some studies included children within narrower age ranges, such as 7–9 years or 5–8 years.

The evaluated dental procedures varied across the studies, ranging from the delivery of local anaesthesia, to pulp therapy, tooth extractions and dental restorations. Some studies focused on specific procedures, such as inferior alveolar nerve blocks, while others assessed intervention effectiveness in a variety of dental procedures, or cooperation at the first dental appointment (Table 7).

**Table 7:** Summary of dental procedures

Article	Dental procedures
**A1** [38]	Composite restorations
**A2** [39]	Infiltration of anaesthetic, pulpotomy and/or restoration of primary first molar
**A3** [40]	Pulpotomy
**A4** [41]	Inferior alveolar nerve block for various dental procedures
**A5** [42]	Short-term dental procedure (< 30 min)
**A6** [43]	Dental procedures not requiring local anaesthesia Painful dental procedures requiring local anaesthesia
**A7** [44]	Topical and infiltrative anaesthesia
**A8** [45]	Primary teeth extraction under local anaesthesia
**A9** [46]	Inferior alveolar nerve block
**A10** [47]	Inferior alveolar nerve block for pulp therapies in primary molars
**A11** [48]	Class I restoration in mandibular primary molars
**A12** [49]	Buccal infiltration local anaesthesia
**A13** [50]	Inferior alveolar nerve block
**A14** [51]	Class I composite resin restoration the mandibular first permanent molar tooth under local anaesthesia
**A15** [52]	Nerve block, extraction or pulpal therapy
**A16** [53]	Dental examination, acclimatization, oral hygiene information, prophylaxis and composite restoration
**A17** [54]	Tooth extraction or dental restorations
**A18** [55]	Pulp therapy treatment
**A19** [56]	Composite restorations
**A20** [57]	Inferior alveolar nerve block for mandibular tooth extraction
**A21** [58]	lnferior alveolar nerve block technique; rubber dam placement
**A22** [59]	Short invasive dental treatment

In this systematic review, different models and brands of VR glasses were observed. Five out of 22 articles included did not specify the VR device used [39, 52, 53, 56, 57], which prevented data comparisons, based on the devices´ specifications, such as size, weight, comfort, or safety indications.

### Results summary

The majority of the studies compared the usage of VR with an alternative technique, during a dental procedure or initial consultation (peri-operatively), except one [39] where the control group included pre-operative exposure to a dental simulation game.

In this systematic review, the primary outcomes were anxiety and pain management in a paediatric dental consultation. In the selected studies, several scales were used for preliminary behaviour assessment and anxiety and pain evaluation, during the dental appointment. It was observed that anxiety was the most investigated aspect, 16 studies [38–41, 44, 46–51, 53, 55–57, 59], while pain perception was addressed in 12 studies [40–43, 45, 49–52, 54, 55, 58, 59]. Scales for anxiety and pain measurement depend on the child´s age and development, hence the variety encountered in the reported studies, as there was a wide age range of participants, from pre-schoolers to pre-teenagers. The referred anxiety scales were CPMAS, MCDAS, MCDAS(f)-r, FIS, VPT and the described pain scales were WBFS, VAS, FLACC, MBPS (Table 8). Some studies also included objective physiological parameters, such as salivary cortisol [40, 59], pulse oximeter [46, 49, 55] and pulse rate [47, 50, 51, 53, 55–57]. In a small number of studies, other aspects were also evaluated, such as fear [42, 45] and cybersickness, nausea and fun [54].

**Table 8:** Measurement scales and protocols

Article	Measurement scales and protocols
**A1** [38]	**Anxiety:** The Children's Perioperative Multidimensional Anxiety Scale questionnaire (CPMAS)
**A2** [39]	**Behaviour:** Frankl’s behaviour rating scale (Patient selection) **Anxiety:** Modified Child Dental Anxiety Scale (MCDAS); Facial Image Scale (FIS)
**A3** [40]	**Behaviour:** Screen for Child Anxiety Related Emotional Disorders **(**SCARED) **Anxiety:** Modified Child Dental Anxiety Scale [MCDAS(f)-r] Faces version; Salivary cortisol levels **Pain:** Wong Baker faces pain rating scale (WBFS)
**A4** [41]	**Behaviour:** Frankl’s behaviour rating scale (Patient selection) **Anxiety:** Modified Child Dental Anxiety Scale (MCDAS) **Pain:** Wong Baker faces pain rating scale (WBFS); Visual Analog Scale (VAS)
**A5** [42]	**Behaviour:** Frankl’s behaviour rating scale (Patient selection) **Pain:** Wong Baker faces pain rating scale (WBFS) **Fear:** Children’s Fear Survey Schedule-Dental Subscale (CFSS-DS)
**A6** [43]	**Pain:** Wong Baker faces pain rating scale (WBFS) Face, Legs, Activity, Cry, Consolability’ scale (FLACC scale); Visual Analog Scale (VAS)
**A7** [44]	**Behaviour:** Frankl’s behaviour rating scale (Patient selection) **Anxiety:** Facial Image Scale (FIS)
**A8** [45]	**Behaviour:** Houpt Scale **Pain:** Wong Baker faces pain rating scale (WBFS) **Fear:** Children’s Fear Survey Schedule-Dental Subscale (CFSS-DS) **Cybersickness:** Simulator sickness questionnaire (SSQ)
**A9** [46]	**Anxiety:** Venham picture test (VPT); Pulse oximeter
**A10** [47]	**Behaviour:** Frankl’s behaviour rating scale (Patient selection); Houpt Scale **Anxiety:** Modified Child Dental Anxiety Scale [MCDAS(f)-r] Faces version; Pulse rate
**A11** [48]	**Anxiety:** Facial Image Scale (FIS)
**A12** [49]	**Behaviour:** Behaviour assessment scale **Anxiety:** Pulse oximeter **Pain:** Wong Baker faces pain rating scale (WBFS); Legs, Activity, Cry, Consolability’ scale (FLACC scale)
**A13** [50]	**Behaviour:** Behaviour assessment scale **Anxiety:** Pulse rate **Pain:** Wong Baker faces pain rating scale (WBFS)
**A14** [51]	**Behaviour:** Frankl’s behaviour rating scale **Anxiety:** Pulse rate **Pain:** Wong Baker faces pain rating scale (WBFS)
**A15** [52]	**Behaviour:** Frankl’s behaviour rating scale (Patient selection) **Pain:** Face, Legs, Activity, Cry, Consolability’ scale (FLACC scale)
**A16** [53]	**Anxiety:** Pulse rate
**A17** [54]	**Pain, quality of the VR experience, nausea and fun:** 0–10 graphic rating scale (Italian scale)
**A18** [55]	**Pain:** Wong Baker faces pain rating scale (WBFS) **Anxiety:** Modified Child Dental Anxiety Scale [MCDAS(f)-r] Faces version; Pulse oximeter and heart rate
**A19** [56]	**Anxiety:** Pulse rate; Facial Image Scale (FIS)
**A20** [57]	**Anxiety:** Facial Image Scale (FIS); Pulse rate and oxygen saturation
**A21** [58]	**Pain:** Wong Baker faces pain rating scale (WBFS); Modified Behavioural Pain Scale (MBPS)
**A22** [59]	**Pain:** Wong Baker faces pain rating scale (WBFS) **Anxiety:** Salivary cortisol levels

Overall, the benefit of VR in controlling anxiety and pain was statistically significant in the included studies, as compared to the corresponding control group, with some exceptions [43, 49, 50, 58]. For instance, VR was comparable to other distraction techniques, such as in Felemban et al. [49], where VR had a similar effect to screen distraction on heart-rate levels and pain during buccal infiltration anaesthesia. In the study by Al-Halabi [50], tablets performed better than VR in relieving anxiety and pain during inferior alveolar nerve block. In two studies, the benefit was not noticed in all the dental procedures, such as in Alshatrat et al. [43], where there was no statistically significant reduction of pain in non-painful dental procedures and Zaidman et al. [58] where VR decreased pain perception during rubber dam placement, but had limited benefit during local anaesthesia.

Özükoç et al. found that children with MIH-affected teeth who are distracted from dental procedures using 3D VR games experienced less dental anxiety (*p* < 0.05) [38]. Concerning short time appointments, Shetty et al., Ran et al. and Kaswindiarti et al., showed a significant reduction in pain, anxiety [40, 42, 59], salivary cortisol (*p* < 0.001) [40, 59] and a shorter treatment time [42]. Regarding delivery of intraoral anaesthesia, MCDAS, VAS and WBFS improved in the immersive VR group [40]. Others presented similar results [39, 45, 47, 50–52, 54, 57]. High levels of satisfaction from children who experienced treatment with 3D video glasses were observed in the study by Nuvvula et al. [47] and increased fun during dental procedures was reported by the participant children in the study of Atzori et al. [54].

## Discussion

This systematic review focuses on comparing the use of VR with conventional non-pharmacological behavioural management techniques in paediatric dental consultations. The selected articles covered various dental procedures such as dental examination, restorations, pulp treatment and anaesthesia. VR was mostly used perioperatively, i.e. simultaneously to treatment delivery. Behaviour, anxiety and pain scales were used to determine efficacy and patient satisfaction. There is strong evidence of the success of VR as a behaviour management tool, in the paediatric dental setting, which in many instances rates superior to conventional behaviour management techniques.

The studies included in this review examined different behavioural control techniques in paediatric dentistry. Conventional techniques were used as a control group in all studies. Some studies used only VR as a test group, while others using a combination of VR with additional techniques like audio, digital screens and smartphone games. Interestingly, none of the studies demonstrated that traditional non-screen techniques were more effective than the tested techniques in reducing anxiety and pain perception. This can be attributed to VR's ability to divert patients' attention to a pleasant virtual environment, thereby modifying the patient perception of physical pain.Three studies A12 [49], A13 [50] and A15 [52] compared the use of digital flat panel devices with VR devices as methods of distraction during local anaesthesia administration. One study A15 [52] found VR devices to be more effective in reducing pain perception compared to other groups. However, two other studies A12 [49] and A13 [50] concluded that tablets provided greater relief from anxiety and pain during anaesthesia. It's important to consider variables that influence children's experiences during dental procedures, such as the type of anaesthesia and the technology (tablet or smartphone) they are familiar with.

Three studies conducted in India A10 [47], A19 [56] and A20 [57] compared the use of VR and audio. Results showed that both audio and VR distraction were effective in reducing anxiety, compared to the conventional "Tell-Show-Do" technique. However, VR proved to be more effective in reducing anxiety and pain perception. While music distraction in the dental environment is widely adopted, VR presents itself as a viable alternative. Only one study A19 [56] included smartphone games alongside VR. It suggested that VR and smartphone gaming were the most effective distraction techniques for managing negative behaviour in paediatric dental patients. When comparing the effectiveness of these techniques, VR distraction was found to be more effective than smartphone game distraction. The VR provided simultaneously an immersive and interactive experience which is likely to have contributed to its greater effectiveness.

One study A4 [41] compared the effect of immersive and non-immersive VR on pain perception during intraoral injections. Both distraction methods were effective in reducing pain perception, with immersive VR slightly more effective. However, the study had limitations such as a small sample size and pain assessment immediately after the injection. Further research is needed to assess the impact of VR distraction in different time points and in a larger sample.

While VR glasses can improve patient cooperation, other factors need to be addressed, such as costs, communication issues, dentists’ perceptions. Some top range VR appliances are expensive; however, prices have become more accessible. VR can also interfere with communication between the dentist and patient during complex procedures, potentially impairing diagnosis and treatment. Vision blockage and absence of caregivers in the visual field can increase children's anxiety [57]. However, one study A17 [54] reported a positive experience of dentists who used VR, feeling more relaxed and focused on their work. Additionally, communication with patients was not affected, despite the use of headsets. Overall, these issues need to be considered when evaluating the use of VR in dentistry.

The increasing use of VR headsets raises health concerns. Prolonged use can lead to eyestrain, dry eyes, vision problems, migraines, dizziness, motion sickness and risk of photosensitive epilepsy. Responsible use of VR headsets is critical to ensure patients' well-being [58, 59] and informed consent needs to be obtained before the use of any VR device on patients..

The appropriate age for using digital equipment, including smartphones and tablets and VR, has been debated, and requires further studies evaluating its long-term effects across different age groups [5, 60]. Immersive media hardware companies have established safety recommendations, with Sony Interactive Entertainment [61], Oculus [62], PlayStation [63] and Samsung [64] stating that their products are not recommended for children under 12 or 13 years old. LG [65] sets the highest age limit at 15, while HTC [66], examined in study A5 [42], has the lowest limit of 4 years without a "safe mode." All articles in this systematic review used VR glasses in children below the manufacturers' recommendations, except for studies without specified equipment brands.

To mitigate adverse effects, researchers have explored strategies such as oculomotor exercises before using VR glasses which have shown effectiveness in reducing cybersickness and associated symptoms. [67]. Taking breaks during VR use is also recommended to prevent digital eye strain, as recommended by the UK Department for Business Energy and Industrial Strategy, in 2020 [68]. However, reviewed studies did not include a specific protocol for preventing eye injuries related to VR glasses.

Assessing cybersickness is crucial as it can cause discomfort and symptoms like nausea, dizziness, headache, eyestrain and general discomfort. It significantly impacts the user experience and may limit the effectiveness of VR applications [58]. However, among the selected articles, only one A8 [45] evaluated cybersickness.

The cost of 3D immersion devices varies based on the type of glasses chosen. Cardboard glasses, the most economical option, use the smartphone screen for display [69, 70]. High-end glasses offer better immersion quality, have their own software and hardware, but still utilize the smartphone as a screen [9]. Gaming glasses are the most expensive and required a computer connection. They are primarily sought after by professional players for superior performance but acquiring them for a dental appointment may not be justified [71, 72]. Overall, VR appears to be a promising behaviour management technique for managing anxiety and reducing pain. However, further studies are needed to compare VR with pharmacological behaviour methods, such as conscious sedation, and to assess its potential for reducing referrals for general anaesthesia. Both VR and pharmacological methods carry a significant financial burden and additional health risks [22, 32].

Video games and digital games served as VR content in some studies [38, 40]. Despite the positive outcomes, patient movement can sometimes interfere with dental examinations or treatments. An emerging application in dentistry is the use of serious games (SGs). These games are increasingly utilized for medical education, training and informative purposes to convey oral health messages [73–75]. In this systematic review, the overall quality of the evidence is good. However, there are certain limitations due to the use of diverse pain and anxiety scales in the included studies which makes direct comparisons difficult. Certain studies included participants with diverse developmental stages, due to the presence of significant age intervals within the sample. Information regarding participants' prior experience with virtual reality (VR) needed to be recorded and participants´ track behaviour during dental appointments was not known. Future research should incorporate qualitative studies to explore patient-reported outcomes and investigate the long-term effects of VR on anxiety and pain. Additionally, details regarding device specifications, screen content and screen time were sometimes omitted or incomplete.

## Conclusion

This systematic review has shown that virtual reality technology during dental treatment is an effective tool for reducing anxiety and pain in children when compared to conventional behavioural management techniques. By creating an engaging and immersive experience, VR successfully shifts the patients' focus away from the clinical environment, resulting in a more positive and enjoyable treatment experience. Therefore, it is crucial that dental professionals become familiar with VR as a valuable tool in the management of paediatric patients. Further research is required to determine the sustained benefits of VR and its integration into routine clinical practice.

### Supplementary Information

**Additional file 1. **Modified Newcastle-Ottawa scale (NOS): Randomised Control Trial. Risk of bias of included studies.

## Data Availability

The datasets used and/or analysed during the current study are available from the corresponding author on reasonable request.

## Abbreviations

PRISMA: Preferred Reporting Items for Systematic Reviews and Meta-Analyses
PICO: Population, Intervention, Comparison, Outcome
MeSH: Medical subject headings
NOS: Newcastle Ottawa scale;
HMD: Head Mounted Devices
FIS: Facial Image Scale:
MCDAS: Modified Child Dental Anxiety Scale:
SCARED: Screen for Child Anxiety Related Disorders;
WBFS: Wong-Baker Faces Pain Rating Scale;
VAS: Visual Analog Scale;
FLACC scale: Face, Legs, Activity, Cry, Consolability’ scale;
VPT: Venham's picture test;
MBPS: Modified Behavioural Pain Scale
SGs: Serious Games
