# Supplementary material for: Virtual reality and behaviour management in paediatric dentistry: a systematic review
Source: BMC Oral Health. 2023 Dec 12;23:995. doi: 10.1186/s12903-023-03595-7 (PMC10717698; doi:10.1186/s12903-023-03595-7)

**Supplementary Material**

**Appendix 1 (.JPG):** Modified Newcastle-Ottawa scale (NOS): Randomised Control Trial

**
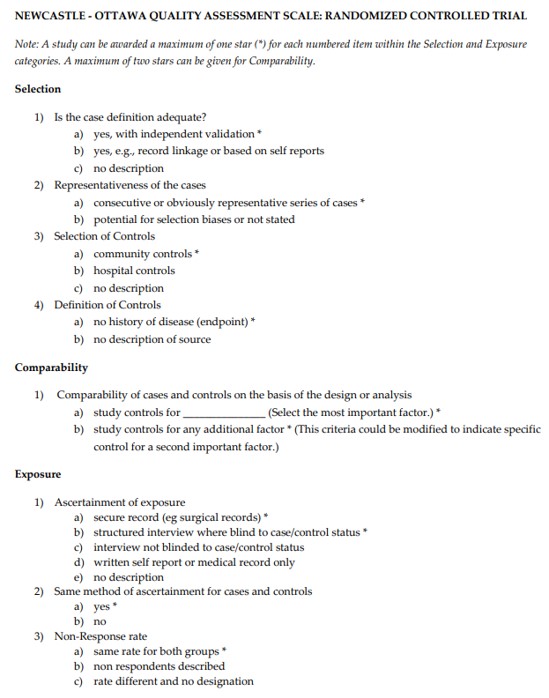
**

**Appendix 2 (.JPG):** Risk of bias of included studies


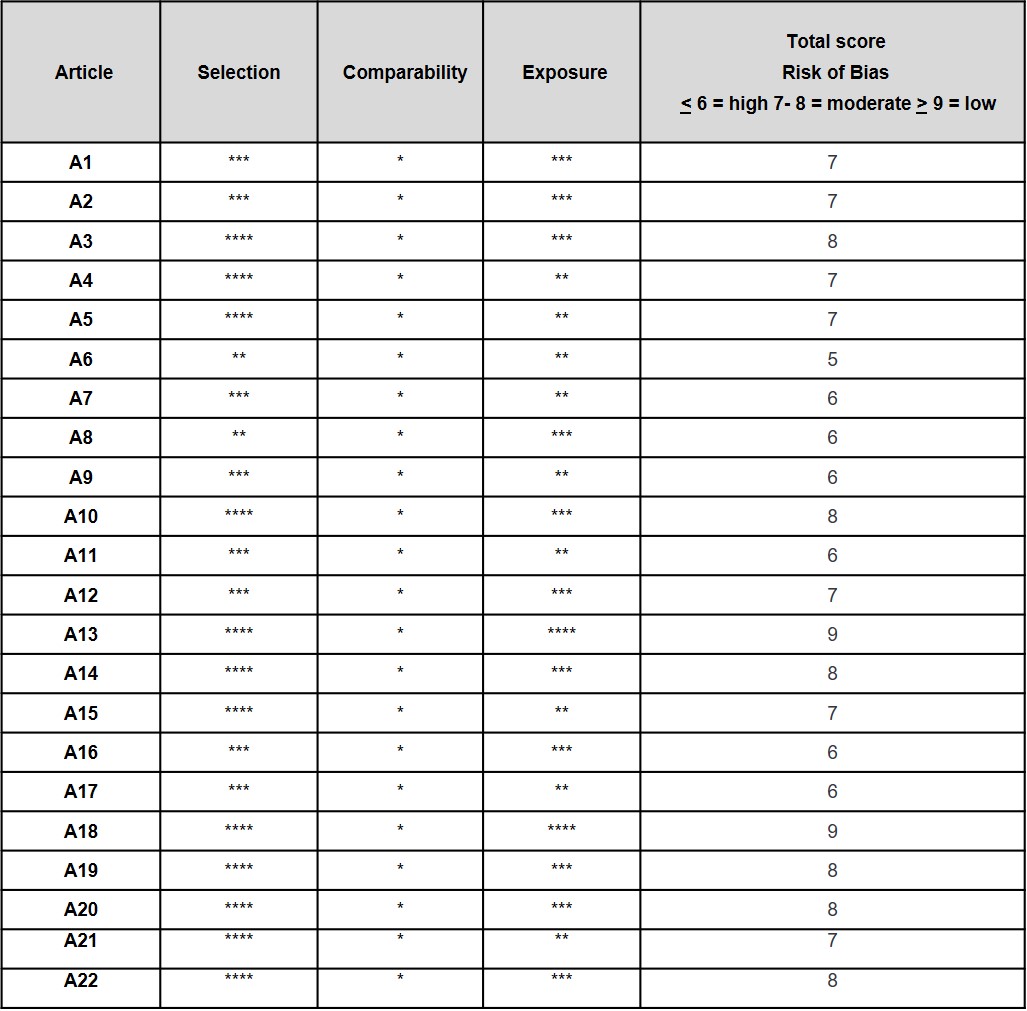

Supplement: Supplementary file 1 — Additional file 1. Modified Newcastle-Ottawa scale (NOS): Randomised Control Trial. Risk of bias of included studies. [file 12903_2023_3595_MOESM1_ESM.docx]
